# Prevalence of diabetic striatopathy and predictive role of glycated hemoglobin level

**DOI:** 10.1007/s10072-022-06304-4

**Published:** 2022-08-04

**Authors:** Silvia Ottaviani, Anna Arecco, Mara Boschetti, Ennio Ottaviani, Paolo Renzetti, Lucio Marinelli

**Affiliations:** 1 Section of Geriatrics, Department of Internal Medicine and Medical Specialties, University of Genova, Genoa, Italy; 2 Endocrinology Unit, Department of Internal Medicine and Medical Specialties, University of Genova, Genoa, Italy; 3 IRCCS Ospedale Policlinico San Martino, Endocrinology Unit, Genoa, Italy; 4 Department of Mathematics, University of Genova, Genoa, Italy; 5 Division of Neuroradiology, IRCCS Ospedale Policlinico San Martino IRCCS, Genoa, Italy; 6 Department of Neuroscience, Rehabilitation, Ophthalmology, Genetics, Maternal and Child Health, University of Genova, Genoa, Italy; 7 IRCCS Ospedale Policlinico San Martino, Division of Clinical Neurophysiology, Genoa, Italy

**Keywords:** Hemichorea, Hemiballism, Diabetes, Striatopathy, Glycated hemoglobin, HbA1c

## Abstract

**Background:**

Diabetic striatopathy is defined as a state of hyperglycemia associated with chorea/ballism, striatal hyperdensity at CT, or hyperintensity at T1-weighted MRI. It is considered a rare complication of uncontrolled diabetes but prevalence data are scarce.

**Objectives:**

Characterize diabetic striatopathy prevalence in the population afferent to the largest teaching hospital in Genova (Liguria, Italy) and investigate the role of glycated hemoglobin level in predicting the risk.

**Methods:**

Data were retrospectively obtained from general population undergoing blood sampling for glycated hemoglobin and resulting with HbA1c values ≥ 8%, from January 2014 to June 2017. Brain neuroimaging of those who underwent at least a brain CT or MRI was examined in search of findings compatible with diabetic striatopathy and clinical information was collected. Logistic regression was used to predict the risk of diabetic striatopathy based on age and HbA1c values.

**Results:**

Subjects with uncontrolled diabetes were 4603. Brain neuroimaging was available in 1806 subjects and three patients with diabetic striatopathy were identified, all of them reporting choreic movements. The prevalence of hemichorea due to diabetic striatopathy was therefore 3 cases out of 1806 (0.16%) in our population. Hepatic and hypoxic encephalopathies were the conditions most frequently mimicking diabetic striatopathy. Odds ratio of diabetic striatopathy and HbA1c level was significantly correlated (*p* = 0.0009).

**Conclusions:**

To the best of our knowledge, this study is the first to evaluate the prevalence of diabetic striatopathy in Italy. High HbA1c values may have a role in predicting diabetic striatopathy.

## Introduction

Diabetic striatopathy is a rare complication of uncontrolled diabetes mellitus that has been known for more than 60 years. It represents an increasingly relevant cause of hemichorea, now second only to cerebrovascular events [1].

Since its first description, made by Bedwell [2], several studies have been published, mainly concerning elderly Asiatic women; in 2002, in fact, according to Oh et al., Asian cases represented 91% of the total [3]. However, in recent years, reports of diabetic striatopathy in other populations are increasing [4–9]. The recent meta-analysis of Chua et al. states that Asia contributes 71.6% of the reported cases, followed by Europe (8.5%) and the Americas (4%) [10].

The clinical importance of this condition is due to the fact that it may represent the first manifestation of an undiagnosed diabetes mellitus, thus allowing a prompt diagnosis and correction of hyperglycemia.

Regarding the nosological definition, we referred to the one reported by Chua et al.: “a state of hyperglycemia associated with at least one of the following conditions: chorea/ballism, striatal hyperdensity on CT scan or hyperintensity on T1-weighted MRI” [10].

Although there are no large epidemiological studies, the prevalence of diabetic striatopathy is reported to be less than 1 in 100,000 [11]; however, this number is likely underestimated because of the lack of knowledge about this condition, which can be misdiagnosed as a striatal hemorrhage. A very interesting epidemiological finding comes from the Mayo Clinic, where Ryan et al. performed a retrospective study; they traced the laboratory and neuroimaging data of all patients presenting with chorea or ballism preceded by hyperglycemia between 2000 and 2014. During this long time span, 596 cases were confirmed as having chorea/ballism, thus returning a prevalence among choreics of 1% [12].

Another interesting finding emerges from a retrospective study carried out by Shafran et al [13]. To define the existence of diabetic striatopathy in the Western population, they reviewed the medical records and neuroimaging of all patients admitted to Chaim Sheba Medical Center (Tel HaShomer, Israel) between 2004 and 2014, identifying those with glycated hemoglobin (HbA1c) > 10%. Of 697 patients, they identified 4 patients (3 women, mean age of 73 and mean HbA1c of 14.8%) with hemichorea or choreoathetosis and brain imaging compatible with diabetic striatopathy (1.2%).

The present work aims to define the prevalence of diabetic striatopathy in the population of uncontrolled diabetics admitted at IRCCS Policlinico San Martino Hospital (Genoa, Italy), in order to provide some preliminary prevalence data. To date, in fact, we are not aware of any other work published to assess the prevalence of striatopathy among diabetics in Italy. In addition, we also tried to explore the possibility of predicting the risk of diabetic striatopathy on the basis of the only data available to us, which are age, sex, and glycated hemoglobin value.

## Materials and methods

The population of interest included uncontrolled diabetics who underwent hemoglobin A1c assessment at the IRCCS Policlinico San Martino Hospital (Genoa, Italy) between January 2nd, 2014, and June 1st, 2017. Specifically, subjects with HbA1c level greater than 8% (64 mmol/L) in at least one assessment were retrospectively included in the study.

The following data were extracted: age, gender, HbA1c values and collection dates, brain CT, and MRI availability.

Brain CT or MRI availability was a prerequisite for further analysis. Unfortunately, many patients had not performed neuroimaging at our Hospital, or imaging was too old and no longer available. We therefore considered only those subjects with at least one available CT or MRI, regardless of whether performed before or after blood collection for HbA1c, and independently on the time interval between neuroimages and altered HbA1c finding. Brain imaging was considered regardless the reason why the CT or MRI was performed (e.g., cerebrovascular disease, trauma).

A two-sample Kolmogorov–Smirnov (KS) non-parametric test was used to investigate if sex, age, and HbA1c were comparable between the subset of subjects with available neuroimaging data and the original population of decompensated diabetics.

We investigated brain CT or MRI availability in relation with subject age to confirm the hypothesis that MRI is performed more often in younger subjects. To this end, the age of subjects who performed MRI and those who did not was compared using a two-sample Student’s *t*-test with 99% significance level. The comparison was repeated in the subsample of subjects with at least one available CT or MRI image, to ensure that data distribution in the reduced sample was comparable to that of the whole original sample of subjects.

The next step was to examine the neuroimages of the patients for whom brain CT or MRI was available, to identify cases with imaging consistent with diabetic striatopathy. Next, we investigated the relationship between affected patients’ demographic and laboratory data and whether neuroimage positivity could be predicted in advance. To this aim, we used logistic regression to model the posterior probability of being positive given sex, age, and HbA1c percentage. The output of the logistic regression was then used and appropriately compared with a threshold value, as a binary classification test for the presence of diabetic striatopathy. As a function of the threshold used, the ROC curve was then evaluated. In this work, we adopt a conservative approach for diabetic striatopathy definition; in fact, we consider affected by diabetic striatopathy only those patients presenting also with hemichorea. Therefore, from now on, “diabetic striatopathy” and “diabetic hemichorea” will be used as synonyms.

## Results

Among 4603 patients identified as uncontrolled diabetic, 1806 had CT and/or MRI images available (39.2%).

The comparison between the whole population and the subset of subjects with neuroimaging data revealed that in the latter group, female gender prevailed and age was higher, while HbA1c levels were comparable with a proved identity at 0.01 significance level (Table 1).

**Table 1:** Distributions of sex, age, and HbA1C in the original population of uncontrolled diabetics, and in the population of patients evaluable for neuroimaging. M: males, F: females, IQR: inter-quartile range, HbA1c: glycated hemoglobin

Parameter	Original distribution	Patients with neuroimaging
Sex	M 55.6% F 44.4%	M 52.9% F 47.1%
Age	Median 69 IQR 18	Median 72 IQR 16
HbA1C	Median 9.0 IQR 1.7	Median 9.0 IQR 1.7

Concerning neuroimaging techniques, CT was consistently prevalent (performed by 95% of patients) compared to MRI (22.5% of patients). The statistical comparison between the age of those who underwent at least one MRI (median 67 years, *IQR* 15 years) and those who did not (median 74 years, *IQR* 17 years) showed that MRI was usually performed in younger patients than CT (*p* = 4 × 10^−26^ estimated by a *t*-test on age data).

Subsequently, based on the presence of striatal hyperdensity on CT and/or hyperintensity on T1-weighted scans, we found 19 patients with basal ganglia lesions compatible with diabetic striatopathy. We verified that all these patients performed neuroimaging within 3 months before high glycated hemoglobin level was disclosed.

**Fig. 1:**
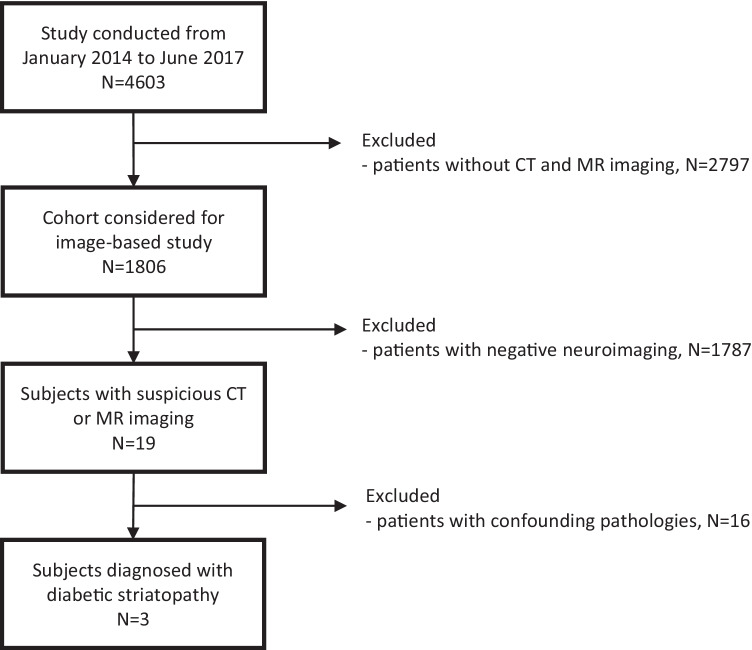
Flowchart of the retrospective study

On further analysis and having retrieved the clinical records, only 3 were found to suffer from diabetic striatopathy. Thus, the prevalence of the disease was 3 cases out of 1806 (corresponding to 0.16%, or 160 cases per 100,000 uncontrolled diabetics) (Fig. 1). The analysis of confounding cases also allowed us to evaluate which may be the most relevant differential diagnoses for striatal hypersignal on CT and T1-weighted scans: hepatic encephalopathy, which manifests with hyperintensity in T1-weighted images, but normal CT findings, and hypoxic encephalopathy, which causes characteristic hyperintense lesions in T2-weighted sequences, diffusion weighted imaging, and in apparent diffusion coefficient maps.

**Fig. 2:**
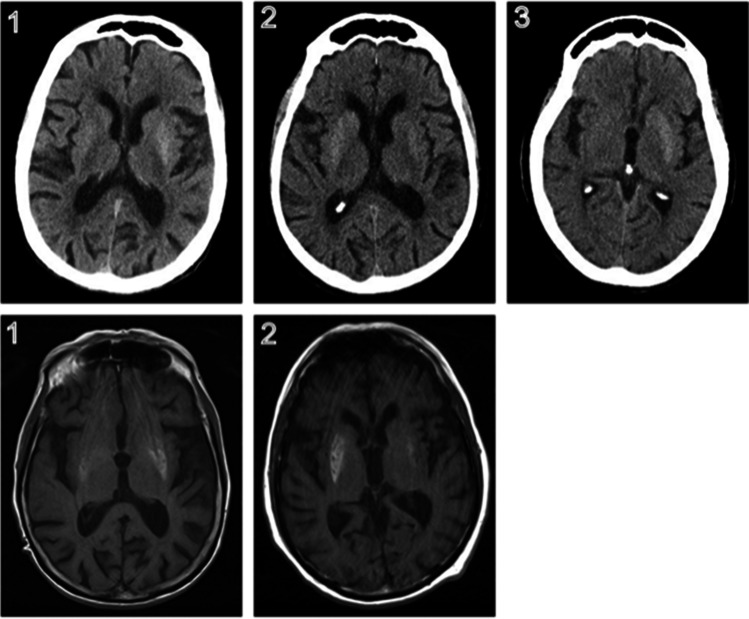
Brain CT (top row) and MRI (bottom row) images of the three patients diagnosed with diabetic striatopathy. The numbers refer to the three patients with diabetic striatopathy who emerged from our analysis

Figure 2 shows CT (top row) and MRI (bottom row) images of the three patients recognized as having diabetic striatopathy. Note that, in contrast to hepatic encephalopathy, where globus pallidus is most frequently affected, in our patients, putamen and caudate nuclei show the prevalent alterations. In particular, thanks to the scrupulous follow-up to which one of the three patients was subjected, we were able to confirm the greater sensitivity of MRI in detecting the disease and its longer latency in negativisation [14].

**Fig. 3:**
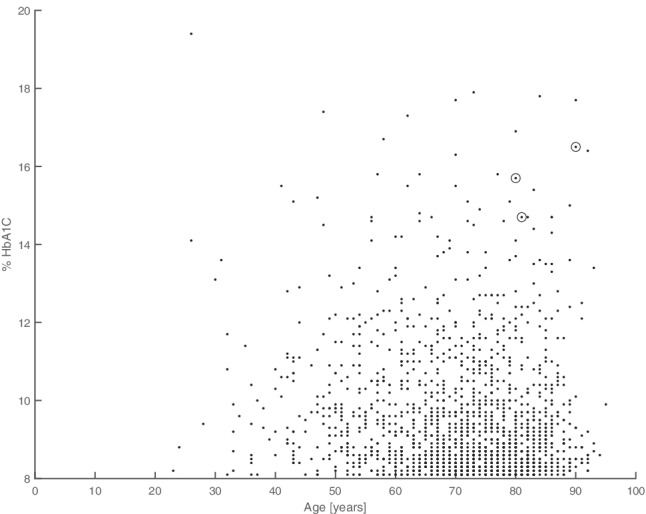
Scatterplot showing HbA1c and age of all analyzed subjects. The HbA1c values of the three patients affected by diabetic striatopathy (circles) lie at the extremes of the distribution

With regard to the attempt to formulate a predictive test, at first, the three subjects were visualized as points on the plane subtended by age and HbA1c value (Fig. 3). Both variables were particularly high: average HbA1c was 15.4% and average age was approximately 84 years.

It should be noted that the three patients are in an extreme area of the plan and can, therefore, be considered outliers with respect to the distribution of diabetic patients. It is therefore reasonable to assume that an unsupervised statistical procedure of automatic anomaly detection could automatically identify these patients among those at higher risk of developing diabetic striatopathy.

Of the two variables, age and HbA1c, the latter has the greatest weight. In fact, looking at the point distribution of Fig. 3, those older than 75 years are still 706 (39% of the total), among whom only 18 have an HbA1c higher than 14%. It follows that the definition of an HbA1c cutoff contributes greatly to effectively narrowing the sample. Logistic regression showed that an explicit linear relationship between the odds ratio of diabetic striatopathy and HbA1c has a highly significant *p* value (0.0009), confirming that the high value observed in positive patients is not due to a random effect. Sex, on the contrary, appears irrelevant for prediction purposes, while the role of age cannot be established with sufficient certainty, at least with the present sample (Table 2).

**Table 2:** Coefficients and *p* values obtained from logistic regression. It can be seen that the correlation with HbA1c value is statistically significant

Parameter	Value	Uncertainty	*p*
Intercept (*a*)	− 27.023	8.3588	0.0012
Sex (β1)	− 0.65871	1.5413	0.6691
Age (β2)	0.13694	0.0911	0.1330
HbA1c value (β3)	0.86282	0.2610	0.0009

**Fig. 4:**
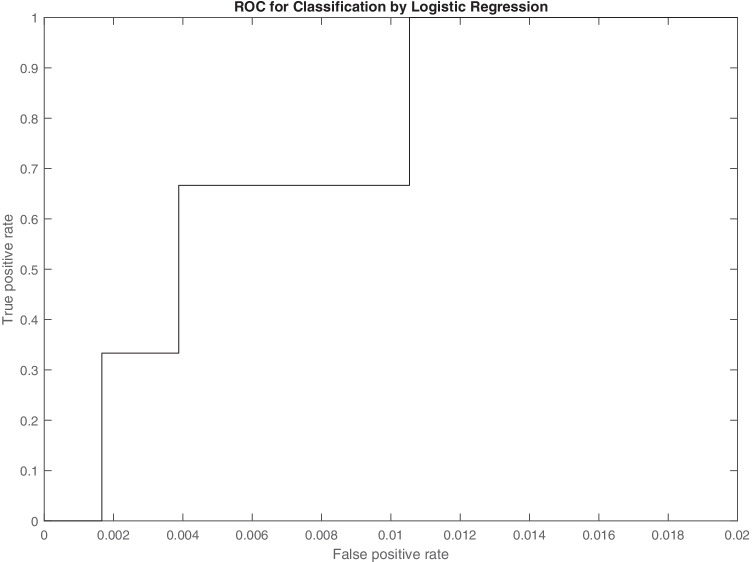
ROC curve (TPR vs FPR) for the binary test of suspected diabetic striatopathy performed on the sample

Subsequently, with a statistical classification test applied to the outcome of the logistic regression, it was possible to obtain the ROC curve (Fig. 4). The test succeeds in correctly classifying all true positives (TPR = 1), while maintaining a very low false positive rate of about 1%. Note that, for reasons of comprehensibility, the x-axis has been restricted to values from 0 to 2% only. The strongly scaled trend of the ROC curve is due, obviously, to the low number of true positives.

## Discussion

As far as we know, this study is the first to provide, with all the limitations arising from the limited sample size, the prevalence of diabetic striatopathy in Italy. The present study also complements the numerous works that have shed light on diabetic striatopathy in ethnic groups other than Asian in recent years [4, 13, 15–17].

The data we obtained (0.16%) reflect a much lower prevalence of diabetic striatopathy than that found by Ryan (1%) and Shafran (1.2%). This can be explained by considering that the target populations are different. Ryan’s study conducted at the Mayo Clinic, in fact, assesses the percentage of patients with diabetic striatopathy out of the total number of patients diagnosed with chorea. Shafran’s study, on the other hand, employs as a denominator all patients admitted to the Chaim Sheba Medical Center between 2004 and 2014 and identifies those with documented elevated HbA1c (> 10%, significantly greater than our cutoff). In contrast, our study reflects a wider and more heterogeneous population, including also totally asymptomatic diabetics admitted through outpatient visits. Furthermore, the prevalence seems to exceed Ondo’s hypothesis (< 1:100,000), probably because it referred to the general population, and not exclusively to uncontrolled diabetics.

In the present study, we demonstrated that knowledge of HbA1c value and age is a good starting point to formulate a reliable prediction of patients at risk of developing this particular form of metabolic hemichorea, although within the limits of the absence of cross-validation. Such finding is somewhat surprising, as we did not expect that age and glycated hemoglobin values alone could take on a predictive role for a complex and still poorly understood phenomenon as diabetic striatopathy. In all probability, in fact, this pathology emerges from the combination of multiple factors: a favorable terrain constituted by the microenvironment of the basal nuclei, made particularly vulnerable to metabolic/hypoxic insults due to their marked metabolic activity; a chronic damage, probably produced by accelerated atherosclerosis, characteristic of diabetics; and finally a triggering factor, perhaps represented by a hyperglycemic/hyperosmolar peak, which determines a strong increase in blood viscosity. CT hyperdensity has been interpreted as the result of petechial hemorrhage following a temporary dysfunction of the blood–brain barrier [18]. T1 hyperintensity, on the other hand, could result from the proliferation of gemistocytes (astrocytes reactive to ischemic insults) [19]. These are only some of the pathogenic hypotheses advanced so far, but many issues remain still open. Hopefully, neuropathology and functional imaging studies will provide answers to the etiopathogenetic hypotheses and early detection of the pathology.

Finally, it should be emphasized that this conclusion is entirely partial and based on a too few cases to be probative. However, this suggestion can provide the basis for further investigations concerning the role of HbA1c value, possibly expressed as a function of age, in the onset of diabetic striatopathy. We acknowledge further limitations in this study. Good practice would require the division of patients into control groups or, in statistical terms, the implementation of the cross-validation procedure. However, we set up a simple screening test for the pathology, in order to apply the direct inspection of CT/MRI images to a smaller subset of patients. The statistical model has also a very low number of free parameters (only 4, including sex) compared to the amount of data available, so the risk of possible overfitting on the data can be considered negligible.

In addition, we did not review the imaging of patients with HBA1c levels below 8% because below this threshold the likelihood of this syndrome is low, but we cannot exclude the possibility that we missed some patients. More importantly, we assessed imaging data independently on the temporal relationship with the timing of HbA1c evaluation; therefore, we cannot be sure about HbA1c levels when neuroimaging was actually performed. It must be said, however, that HbA1c reflects glycemia over a time span of about 3 months and levels above 8% probably indicate a long lasting decompensated status. Since only 39.2% of decompensated diabetic patients performed brain neuroimaging and also considering that the time interval between glycated hemoglobin dosage and neuroimaging was variable, it is likely that the prevalence of diabetic striatopathy in our sample is underestimated.

In conclusion, diabetic striatopathy is a rare complication of uncontrolled diabetes mellitus. However, in relation to the increasing incidence of diabetes, it is now reported to be the second most frequent cause of hemichorea, after cerebrovascular events. The objective of research on this disease is not, therefore, purely speculative; the dissemination of a wider knowledge of diabetic striatopathy, in fact, would allow to detect the condition early, immediately establish the correct treatment, and promptly resolve the symptoms in most cases.

In addition, the outcome of the preliminary predictive test advanced by us, based on age and glycated hemoglobin value, opens the way to new insights into the screening of diabetic complications.
